# PTSD Symptom Severity Associated With Sleep Disturbances in Military Personnel: Evidence From a Prospective Controlled Study With Ecological Recordings

**DOI:** 10.1155/da/8011375

**Published:** 2025-04-25

**Authors:** Emeric Saguin, Dorone Feingold, Gilles Sipahimalani, Michael Quiquempoix, Jean-Baptiste Roseau, Marion Remadi, Sophie Annette, Mathias Guillard, Pascal Van Beers, Bertrand Lahutte, Damien Leger, Danielle Gomez-Merino, Mounir Chennaoui

**Affiliations:** ^1^Department of Psychiatry, Begin National Military Teaching Hospital, Saint-Mandé, France; ^2^UMR VIFASOM (Vigilance, Fatigue, Sleep and Public Health), Université Paris Cité, Paris, France; ^3^Department of Psychiatry, Percy National Military Teaching Hospital, Clamart, France; ^4^French Armed Forces Biomedical Research Institute, Brétigny-sur-Orge, France; ^5^Department of Pulmonology and Sleep Medicine, Clermont-Tonnerre Regional Military Teaching Hospital, Brest, France; ^6^Val-de-Grâce French Military Medical Academy, Paris, France; ^7^Sleep and Vigilance Center, Hôtel-Dieu, AP-HP, Paris, France

**Keywords:** military personnel, PTSD, sleep, trauma-related nightmares, wearable devices

## Abstract

Sleep disturbances, including insomnia and trauma-related nightmares (TRNs), are the core symptoms of post-traumatic stress disorder (PTSD) in military personnel. Furthermore these nocturnal manifestations are directly related to the persistence of daytime PTSD symptoms and are known to exacerbate comorbid conditions such as depression, suicidality, and daytime impairments. This prospective study examined the variability of PTSD-related sleep disruptions and its relationship to symptom severity using ecological recordings over several nights. One hundred thirty PTSD-diagnosed service members and 65 healthy military controls recorded sleep data at home for five nights using a polysomnographic headband to measure total sleep time (TST), sleep onset latency (SOL), wake after sleep onset (WASO), sleep efficiency index (SEI), and sleep stages. PTSD severity and comorbid symptoms were assessed by clinical evaluations. Compared to controls, PTSD participants had higher SOL and WASO (+14.1 min and +9.1 min, *p* < 0.001, respectively), reduced SEI (−6.6%, *p*  < 0.001), and lower N3 and rapid eye movement (REM) sleep durations. In addition, night-to-night variability (NNV) in SOL and WASO was higher in the PTSD group. The sleep fragmentation index (FI)—and more specifically non-REM (NREM) sleep fragmentation—was significantly correlated with PTSD severity, particularly the intrusive and avoidance symptoms clusters in the PCL-5 score. The results highlight the need for customized multinight assessments to study sleep variability in military patients with combat-related PTSD, in order to advance therapeutic strategies for military populations.

**Trial Registration:** ClinicalTrials.gov Identifier: NCT04581850

## 1. Introduction

The military population is at risk of sleep disturbances due to occupational demands, leading to short sleep duration [1]. Although sleep disturbances are frequently regarded as consequences of post-traumatic stress disorder (PTSD), growing evidence suggests that these nocturnal manifestations not only contribute causally to the onset and persistence of PTSD, but are also directly related to the continuation of daytime symptoms and the exacerbation of comorbidities such as depression, suicidality, and daytime impairments [2]. Prospective studies in soldiers indicate that sleep alterations, both before and after trauma exposure, predict PTSD onset, reinforcing the hypothesis that disturbed sleep contributes to PTSD vulnerability [3, 4].

In line with these findings, sleep disturbances are among the most frequently reported symptoms in PTSD, affecting between 70% and 90% of military personnel with the disorder [4–6]. In veterans with war-related PTSD, 74% report sleep complaints, including difficulty falling asleep, frequent nocturnal awakenings, and poor sleep efficiency. Their estimated total sleep time (TST) is around 5 h per night, significantly lower than in the general population [4, 7]. Insomnia is particularly prevalent, reported in 92% of cases before treatment and persisting in 74%–80% of patients afterward [8]. Trauma-related nightmares (TRNs) are also common, affecting 69% of patients before treatment and remaining in 49%–55% of cases [8]. Despite treatment, these disturbances frequently persist, reinforcing their role in PTSD chronicity [2, 8–10].

Findings from several studies suggest that sleep fragmentation—characterized by frequent micro-awakenings and unstable sleep architecture—may be a key mechanism linking sleep disturbances to PTSD symptomatology. Observed both before combat-related trauma and in the acute post-trauma phase, sleep fragmentation has been proposed as a potential predictive marker for the development of chronic PTSD [10–12]. Moreover, significant correlations have been shown between sleep instability and the specific PTSD symptoms [13], reinforcing this potential mechanistic link. Although these mechanisms remain hypothetical, sleep fragmentation—particularly during rapid eye movement (REM) and Non-REM (NREM) sleep—has been suggested as a key factor in PTSD-related dysfunctions. While REM sleep fragmentation may impair the emotional processing of traumatic memories, NREM sleep disturbances, particularly deep sleep reduction, could hinder initial memory stabilization and adaptive processing of traumatic experiences [14, 15]. These alterations were associated with deficits in fear extinction, emotional memory processing, and increased frequency of TRNs, supporting the idea that sleep dysfunctions might reinforce PTSD symptomatology rather than being mere correlates of it [14, 16].

While the study and clinical management of sleep disturbances in PTSD are critical, they remain challenging. A meta-analysis of 31 polysomnographic studies identified consistent alterations in sleep architecture and continuity in PTSD patients compared to healthy controls [14]. Results indicate that PTSD is associated with a lower sleep efficiency index (SEI; 80% in patients vs. 83% in controls), increased sleep onset latency (SOL), more frequent wake after sleep onset (WASO), reduced TST, a lower percentage of N3 (deep sleep), and a lower percentage of REM sleep only in patients with a mean age below 30 years [14]. The REM sleep findings remain inconsistent across studies, with some reporting reduced REM duration, while others find no significant change or even increased REM density [14]. Such discrepancies may be due to differences in sample characteristics, such as trauma type or gender, highlighting the need for more detailed demographic and clinical information in the research to ensure comparability between patient and control groups [11, 17–19]. In addition, the methods used to record sleep are another factor that could explain variations in measurements and the lack of specific response patterns.

Although the gold standard, polysomnography (PSG) often fails to reflect the sleep patterns of PTSD patients due to the artificial environment (i.e., sleep laboratory) and fixed schedules, which can mask specific sleep disturbances and fail to match patients' complaints [20]. For instance, PTSD patients report fewer TRNs in the sleep lab compared to their home environment, where subjective complaints are more pronounced [18, 21]. Furthermore, a single-night PSG recording is insufficient to assess night-to-night variability (NNV) in sleep disturbances, particularly those with large fluctuations related to daily variations in PTSD symptoms [20, 22]. This limitation is illustrated by a study that found greater intra- and interindividual variability in sleep disturbances among PTSD veterans compared to those with primary insomnia and healthy controls [23].

To overcome these methodological challenges, researchers are increasingly turning to multinight home sleep recordings or hybrid approaches that combine laboratory and ecological assessments [21, 24, 25]. Our recent study used a headband electroencephalography (EEG) device to record the sleep of military personnel suffering from PTSD, yielding a large amount of data and establishing a more ecologically valid correlation between subjective and objective sleep parameters [25].

Given the high prevalence and significant impact of sleep disturbances on PTSD symptoms, it is important in sleep medicine to refine assessment methods to better capture their real-life manifestations. It is essential to develop methodological approaches in ecological settings, using wearable recording devices, in order to more accurately document sleep disturbances and their variability in PTSD. This will not only improve measurement precision but also deepen our understanding of the interaction between sleep alterations and PTSD severity and ultimately inform targeted interventions.

## 2. Aims

In this report, our primary objective was to characterize the clinical features of veterans and active-duty military personnel with severe PTSD and to compare their home-recorded sleep patterns with those of healthy military controls. Sleep recordings were collected over several consecutive nights at home, enabling an ecologically valid assessment of sleep [25]. We hypothesized that PTSD patients would exhibit reduced sleep quality and quantity (i.e., lower SEI and TST) consistent with previous reports of fragmented and insufficient sleep in PTSD.

A secondary objective was to assess within-group and NNV in sleep parameters. Given previous evidence of sleep instability in PTSD, we hypothesized that patients would show greater fluctuations in key parameters such as TST, WASO, SOL, and SEI compared to controls.

Finally, we examined associations between home-recorded sleep parameters, subjective sleep ratings, and PTSD severity in two steps. First, we assessed the correlation between objective sleep measures (TST, WASO, SOL, and TRNs) and self-reported sleep quality, assuming that a strong correlation would support the clinical validity of our recordings. Second, we assessed the relationships between objective sleep parameters and PTSD severity (i.e., the four symptoms clusters of the PCL-5 scale). We hypothesized that increased sleep fragmentation, prolonged WASO and SOL, and reduced TST would be related to the intrusive symptom (Cluster B; including TRNs), while reduced TST and increased fragmentation would be associated with hyperarousal symptoms (Cluster E).

## 3. Methods

### 3.1. Participants

#### 3.1.1. Patients With PTSD (PTSD-G)

The study included 130 veterans and active-duty service members, all suffering from PTSD and treated at five French military hospitals between November 2020 and March 2023. This clinical research on sleep in PTSD patients received ethical approval (reference number 2020-80) from the Committee for the Protection of Persons (CPP), Southeast 1 (EUDRACT 2020-A01808-31, RIPH2). Recruitment was conducted through flyers and referrals from psychiatrists at military hospitals.

Inclusion criteria were: (a) veterans or active-duty military personnel, (b) receiving treatment in the psychiatric department of a military hospital, and (c) diagnosed with combat-related PTSD (PCL-S > 44 and a clinical diagnosis of PTSD). Complaints of sleep disturbances or nightmares were not required for inclusion. Exclusion criteria prior to the trauma included: (a) a history of chronic psychiatric disorders, (b) identified sleep disorders, and (c) neurological disorders.

#### 3.1.2. Healthy Military Control Group (HM-G)

The control group consisted of 65 healthy military personnel recruited from several Army units. Recruitment was conducted via flyers or recommendations from their physicians. Participants underwent the same evaluations as PTSD patients. The exclusion criteria were as follows: (a) a history of chronic psychiatric disorders including PTSD, (b) identified sleep disorders, and (c) neurological disorders.

#### 3.1.3. Healthy Civilian Group (HC-G)

We also used data from a group of 52 healthy civilians to provide a reference for the sleep recordings and to ensure that the military control group was representative of healthy subjects. Clinical data and sleep analyses for this group were supplied by Dreem from their proprietary database. Sleep was recorded at home using the Dreem 2 headband (DH) [26].

### 3.2. Procedure

After signing the informed consent form, PTSD-G and HM-G underwent a series of clinical assessments before receiving the sleep recording equipment. They were asked to return it after 1 month and undergo the same clinical assessments again. The detailed protocol of this study has been described previously [25].

#### 3.2.1. Clinical Assessment

All biographical data, military history, trauma and medical history, medications, and PTSD diagnosis confirmation were collected by an experienced clinician during a semistructured interview using a case report form (CRF) developed specifically for this study by a consortium of military psychiatrists and researchers. PTSD assessments included the PCL-S scale [27], the PCL-5 scale [28], and the impact of event revised scale (IES-R) [29]. Depression was assessed using the Beck depression inventory (BDI) [30]. Sleep characteristics were evaluated using the Pittsburgh sleep quality index (PSQI) [31], the PSQI addendum (PSQI-A) for PTSD [32], the TRN survey-French version (TRNS-FR) [33], and the insomnia severity index (ISI) [34]. The risk of obstructive sleep apnea (OSA) was assessed using the Berlin questionnaire [35]. Daytime sleepiness was evaluated using the Epworth sleepiness scale (ESS) [36].

**Figure 1:**
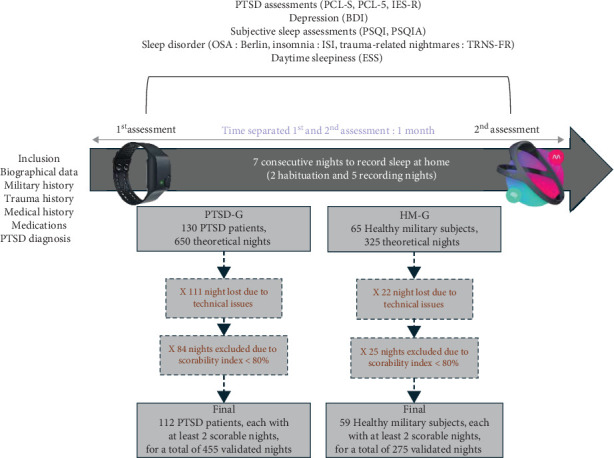
Study timeline and selection process for recorded nights.

Participants completed questionnaires during both the first and second assessment periods. We primarily used data from the second assessment, as these questionnaires capture experiences from the preceding weeks. When necessary, data from the first assessment were used to ensure completeness. A schematic timeline of the sleep and clinical assessments is shown at the top of Figure 1.

#### 3.2.2. Electrophysiological Recordings

After they received standardized training on how to use it, PTSD-G and HM-G were equipped with the DH for sleep recording and the Empatica E4 wristband (E4) to signal TRNs. A summary of these instructions was then lent to them for 1 month during which they were asked to choose seven consecutive nights to record their sleep. The first two nights were devoted to habituation and the last five nights to recording. After 1 month, they returned the equipment and underwent the same assessment as inclusion.

• The DH is a validated alternative to PSG that offers user-friendly sleep stage recording with comparable accuracy to experienced scorers [26]. Unlike PSG, which is typically conducted in a sleep clinic, the DH device allows nightly use at home. This wireless headband records, stores, and analyzes physiological data in real-time without requiring external connections. Sleep stages are objectively measured using built-in sensors, including five EEG dry electrodes providing seven EEG channels (FpZ-O1, FpZ-O2, FpZ-F7, F8-F7, F7-O1, F8-O2, FpZ-F8; sampling rate of 250 Hz with a 0.4–35 Hz bandpass filter), a 3-D accelerometer positioned over the head to track movements, position, and breathing frequency, and a red-infrared pulse oximeter in the frontal band to record heart rate. In a cohort of healthy subjects, DH demonstrated automated sleep staging classification in accordance with the American Academy of Sleep Medicine (AASM) criteria, showing performance equivalent to the consensus scoring by five medical-grade PSG scorers [26].
• In this study, we employed the automated algorithm integrated into the DH, which functions in two stages: data extraction and classification. During the data extraction stage, 30-s epochs of sensor data are processed to capture EEG power frequencies and identify sleep patterns, utilizing temporal context to predict current epochs based on previous ones. The classification stage then applies two layers of long–short term memory (LSTM) networks, followed by a Softmax function, to produce the final probability prediction for sleep stage classification [26].
• The E4 wristband (E4) is a lightweight device that enables continuous recording of several physiological signals and features a push-button allowing patients to report events that occur during the night. It is validated as a medical device and complies with the CE Medical 93/42/EEC Directive, Class 2A [37]. In this study, E4 was used solely to objectively measure the frequency of TRNs reported at home. The instructions given to the participants were based on a standardized script that was read to them during initial assessment and briefing (‘If, during the night, you wake up in the aftermath of a TRN, that is, a sudden awakening associated with the memory of an anxiety-provoking nightmare, you can report it by a brief pressure (a simple “click”) on the button of the wristband') [25].

### 3.3. Sleep Analysis

A minimum of two nights of home recording was fixed for inclusion in the sleep analyses. Sleep analyses were conducted using a range of metrics to comprehensively describe the recordings. Various sleep parameters were assessed, including TST, SOL, WASO, SEI (TST/time in bed), and total wake time duration.

Sleep stages were analyzed by measuring the duration and percentage of time spent in each stage: N1, N2, N3, REM, and combined NREM ( = N1 + N2 + N3) sleep. Latencies to specific sleep stages were also recorded, including latency to N2, N3, and REM.

To assess sleep fragmentation, we calculated the fragmentation index (FI; events per hour) [38], then, the REM and NREM sleep fragmentation indices (events per hour) using the same methodology and finally the microarousal index. All these parameters were calculated in accordance with the recommendations of the AASM. These parameters provided a detailed profile of sleep architecture and quality, enabling an in-depth examination of sleep disturbances in the populations studied.

To investigate the second hypothesis, which suggests greater sleep variability among PTSD-G, NNV and within-group variability (WGV) were calculated for PTSD-G and HM-G, following the methodology described by Straus et al. [23]. NNV was used to assess the consistency of sleep parameters (TST, SOL, WASO, and SEI) from night to night, while WGV captured their variability within a group over time. The root mean squared successive difference (RMSSD) values were calculated and served as the index for NNV; the variance differences between the groups were analyzed for WGV.

### 3.4. Statistical Analyses

We initiated the analysis by conducting a descriptive examination of all sleep parameters in PTSD-G, comparing these patterns with those of a HM-G under identical conditions. Descriptive analyses included the number of occurrences (*N*), percentage (%), mean, and, where necessary, standard deviation (SD). Differences between values were analyzed using a one-way repeated measures ANOVA. When the normality assumption was violated, the Kruskal–Wallis nonparametric test was used with effect sizes reported using epsilon squared (*ε*^2^) and categorized as small (*ε*^2^ < 0.01), medium (0.01 ≤ *ε*^2^ < 0.10), or large (*ε*^2^≥0.10). For sleep parameters, an analysis of covariance (ANCOVA) was conducted, with age and treatment included as covariates to control for their potential confounding effects. Multiple comparisons were addressed by employing the Benjamini–Hochberg procedure, with the false discovery rate (FDR) set at 5%. Adjusted *p*-values below 0.05 were considered statistically significant. For *p*-values reported as “<0.001,” a value of 0.001 was used in the analyses.

For NNV, a bootstrapping procedure, which involved drawing 250 samples with replacement, was used to statistically compare the two groups. This approach generated a sampling distribution of differences in RMSSD between groups, yielding standard errors and 95% confidence intervals (CIs). Statistical significance was established when the 95% CIs did not encompass zero. Additionally, we used the Mann–Whitney *U* test to assess the significance of NNV differences. For WGV, variance differences between the groups were analyzed using the same bootstrapping method and a nonparametric repeated-measures ANOVA (Friedman test), as the data did not meet normality assumptions. In our analyses of NNV and WGV, a Bonferroni correction was applied to adjust for the four tested variables. Consequently, the significance threshold was set at *p*  < 0.0125 to control for type I errors.

Finally, to statistically examine the relationship between home-recorded sleep data and clinical measures of PTSD symptomatology, including sleep disturbances and associated symptoms, we performed a correlation analysis. This was performed using Spearman's correlation coefficient (*ρ*) for nonparametric data. Spearman's coefficient ranges were interpreted as follows: 0.20–0.29 for weak correlations, 0.30–0.39 for moderate correlations, 0.40–0.69 for strong correlations, and >0.70 for very strong correlations [39]. The normality of data distribution was verified using the Shapiro–Wilk test. For the correlation matrix, 40 tests assessing associations between eight sleep variables and five PCL-5 scores were corrected for multiple comparisons using the Benjamini–Hochberg procedure to control the FDR at 5%. *p*-values were ranked in ascending order and adjusted according to their rank and the total number of tests. Corrected *p*-values below 0.05 were considered statistically significant, thereby, balancing type I error control with statistical power.

Statistical procedures were performed using Jamovi (version 1.6.23; computer software; retrieved from https://www.jamovi.org, Sydney, Australia) and MATLAB release 2024a for specific data analyses.

## 4. Results

### 4.1. Sociodemographic Characteristics

One hundred thirty patients suffering from PTSD were included in PTSD-G. Of these, 125 were male and five were female, with a mean age of 39.9 years (SD = 9.3). At the time of the study, 78.1% of the patients were still active-duty military personnel, with an average length of service of 17.7 years (SD = 8.3). At inclusion, 70.9% of patients were on sick leave. On average, each patient had participated in 5.15 overseas deployments (SD = 4.45) and accumulated 18.1 months (SD = 18.3) in such operations. These patients had an average history of 7.07 traumatic events (SD = 11.71). The most frequent events included encounters with death (92.2%), witnessing injuries (62.5%), combat actions (55.47%), and exposure to explosions (50.78%). They were diagnosed with PTSD approximately 3.42 years prior to the study (SD = 2.68).

The HM-G group, recruited from army regiments, consisted of 59 males and six females, with an average age of 35.20 years (SD = 8.93). On average, they had participated in 4.63 overseas deployments (SD = 3.71) and accumulated a total of 17.18 months (SD = 13.35) in operations. Thirty-nine (60.00%) had experienced at least one traumatic event during a deployment.

The HC-G group, consisting of 52 individuals (49 men and three women; mean age 43 years, SD = 8.70), was included primarily to validate the sleep recording method used for the HM-G group and to ensure consistency in the results. As such, this group will not be further described in detail.

### 4.2. Clinical Description

PTSD-G patients presented severe PTSD, as indicated by an average PCL-5 score of 46.72. Additionally, 98.37% of the patients experienced depressive episodes, with 35.77% classified with severe depression, 47.15% with moderate depression, and 15.45% with mild depression. All patients had a total PSQI score greater than 3, indicating the presence of significant sleep disturbances. Most frequent sleep complaints were about TRNs (84.25%) and increased SOL (70.08%). According to the Berlin questionnaire, 64.23% of patients were identified as high risk for OSA. There was a significant difference between PTSD-G and HM-G in all these clinical parameters. All these results remained significant after applying the Benjamini–Hochberg procedure for multiple comparisons. These results are presented in Table 1.

Regarding substance use in PTSD-G, 46.09% of patients reported regular alcohol consumption, averaging 13.2 units per week. Five reported using cannabis, two reported using CBD, and one reported using cocaine. In terms of prescribed psychotropic treatments, only 21 patients (15.15%) were not receiving any medication. Eighty-two patients (63.08%) were receiving antidepressants, 31 (23.85%) were prescribed long-term antipsychotic medication, and 49 (37.69%) were receiving short-acting antipsychotics, primarily loxapine (10–30 mg at bedtime). Thirty patients (23.08%) had a prescription for benzodiazepines and 22 patients (16.92%) were receiving hypnotics. Other prescribed treatments included hydroxyzine (14.62%), mood stabilizers (6.92%), alimemazine (5.70%), and prazosin (5.33%). Overall, 55.9% of the patients were receiving treatment specifically targeting sleep, such as short-acting sedatives or hypnotics to be taken before bedtime. Five patients had been diagnosed with OSA and received appropriate treatment (continuous positive airway pressure or mandibular advancement device). None of the patients in the HM-G group were undergoing any treatment.

### 4.3. Sleep Recordings

#### 4.3.1. HM-G vs. HC-G (Control Groups)

To ensure that the HM-G was representative of a healthy population, we compared it to the HC-G group which is made up of regular DH users. Of the 65 subjects in HM-G, only 59 were able to complete the home recordings, that is, 90.8%. They recorded a total of 275 nights (Figure 1), with an average of 4.66 nights per subject (SD = 0.63). In comparison, the 52 civilians in the HC-G recorded a total of 230 nights, with an average of 4.42 nights per subject (SD = 0.96).

There were no significant differences between the HM-G and HC-G groups for the sleep parameters, except for TST (*χ*^2^ = 5.0429, *p*=0.025, *ε*^2^ = 0.0458) and FI (*χ*^2^ = 8.6610, *p*=0.003, *ε*^2^ = 0.0787). The mean TST was 421.79 (SD = 48.42) minutes for the HC-G group and 399.79 min (SD = 53.31) for the HM-G group, while the mean FI was 6.71 (SD = 1.36) for the HC-G group and 5.94 for the HM-G group. However, after correction for multiple comparisons using the Benjamini–Hochberg procedure, these differences did not remain significant (adjusted *p*-value respectively of 0.005 and 0.0025).

#### 4.3.2. PTSD-G vs. HM-G

Of the 130 patients in the PTSD group, 112 met the minimum requirement of recording at least two nights for inclusion in the study, representing approximately 86.15% of the participants. On average, these patients recorded 455 nights (Figure 1) equivalent to an average of 4.06 nights per patients (SD = 1.08). Of the 130 patients in the PTSD group, 71 (54.62%) reported at least one TRN during the study. Within this subgroup, 347 TRNs were recorded over 162 nights. When extrapolated to a 7-day period, this equates to approximately 8.93 TRNs per week per patient.

Significant differences were observed between PTSD-G and HM-G in several sleep parameters, including SOL (*χ*^2^ = 28.66, *p*  < 0.001, *ε*^2^ = 0.1686), WASO (*χ*^2^ = 13.079, *p* < 0.001, *ε*^2^ = 0.0769), SEI (*χ*^2^ = 30.08, *p*  < 0.001, *ε*^2^ = 0.0769), wake duration (*χ*^2^ = 29.181, *p* < 0.001, *ε*^2^ = 0.1717), N3 sleep duration (*χ*^2^ = 5.8443, *p*=0.016, *ε*^2^ = 0.0344), REM sleep duration (*χ*^2^ = 5.9469, *p*=0.015, *ε*^2^ = 0.0350), N2 sleep percentage (*χ*^2^ = 9.1907, *p*=0.002, *ε*^2^ = 0.0541), REM sleep percentage (*χ*^2^ = 8.7047, *p*=0.003, *ε*^2^ = 0.0512), and NREM sleep percentage (*χ*^2^ = 8.7047, *p*=0.003, *ε*^2^ = 0.0512). Additionally, there were significant differences in the latencies of N3 sleep (*χ*^2^ = 8.0376, *p*=0.005, *ε*^2^ = 0.0473) and REM sleep (*χ*^2^ = 12.2696, *p*  < 0.001, *ε*^2^ = 0.0722).

All these results remained significant after applying the Benjamini–Hochberg procedure for multiple comparisons.

Due to significant age differences between PTSD-G and HM-G, we conducted an ANCOVA to adjust the results for age and the presence of psychotropic treatment. While interactions for SOL duration, wake duration, SEI, and N3 sleep latency remained significant, the other parameters were no longer significant. Results are presented in Table 2.

#### 4.3.3. WGV

Significant differences were observed for SOL, WASO, and SEI between the PTSD-G and the HM-G, while no significant differences were observed for TST.

For SOL, the PTSD-G showed a higher median and greater variability compared to the HM-G, highlighting that individuals with PTSD took longer and exhibited more variability in the time required to fall asleep. This was supported by a WGV 95% CI of (330.8161, 946.4712) min^2^; a corrected *p*-value from the Friedman test of <0.001; and a chi-square value of *χ*^2^ = 0.547.

For WASO, the PTSD-G exhibited a higher median and greater variability, with more outliers indicating increased frequency and duration of wakefulness during the night. In contrast, the HM-G had lower WASO and less variability, indicating improved sleep continuity (WASO WGV 95% CI: (160.189, 587.9676) min^2^; *p*  < 0.001; *χ*^2^ = 15.4).

SEI was also significantly lower and more variable in the PTSD-G, with a lower median and a wider spread, while the HM-G had a higher median SEI with fewer outliers and less variability, indicating better sleep efficiency (SEI WGV 95% CI of (28.8342%, 75.5045%), *p*  < 0.001, *χ*^2^ = 51.5).

Although the TST was lower on average for the PTSD-G, with a wider spread and more outliers, compared to HM-G, which had a higher median TST with a narrower distribution, no statistically significant difference was found. The WGV 95% CI of (555.04801, 4077.9757) min^2^ indicated a trend toward significance; however, the corrected *p*-value from the Friedman test showed no meaningful difference (*p*=0.902, *χ*^2^ = 0.619).

**Figure 2:**
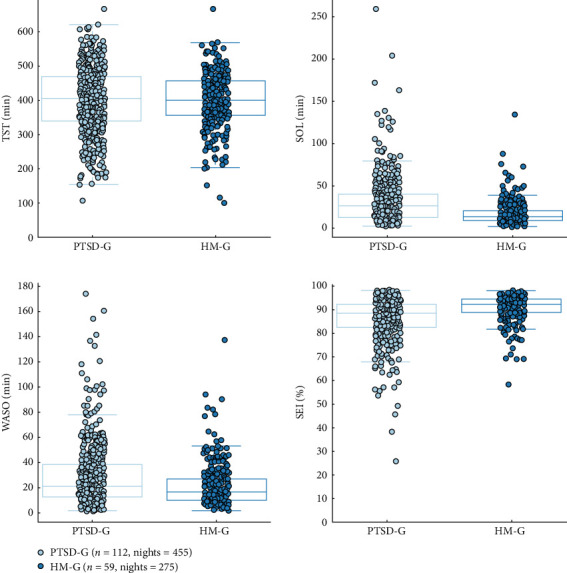
Boxplots displaying the distribution of the different sleep parameters for within-group variability (WGV) between PTSD-G (*n*_Subjects_ = 119, *n*_Nights_ = 455) and HM-G (*n*_Subjects_ = 59, *n*_Nights_ = 275). *⁣*^*∗∗∗*^*p*  < 0.001, statistical differences between the two groups.

These results are illustrated in Figure 2.

#### 4.3.4. NNV

Significant differences were found for SOL, WASO, and SEI, while no significant differences were detected for TST.

For SOL, the HM-G exhibited lower RMSSD values, reflecting reduced variability compared to the PTSD-G, with a 95% CI of (3.537, 15.322) minutes and a corrected *p*-value of 0.002, confirming statistical significance. A similar pattern was observed for WASO, where the HM-G also showed less variability, supported by a 95% CI of (2.616, 11.495) minutes and a corrected *p*-value of 0.005, indicating a significant difference. Additionally, SEI demonstrated significantly lower variability in the HM-G compared to PTSD-G (95% CI (0.325%, 3.723%), with corrected *p*-value = 0.0004).

In contrast, analysis of the TST variability revealed no significant difference between the groups. While the HM-G exhibited a wider RMSSD distribution, the 95% CI of (−15.817, 16.085) minutes and a corrected *p*-value of 0.806 indicated no meaningful difference in TST variability.

**Figure 3:**
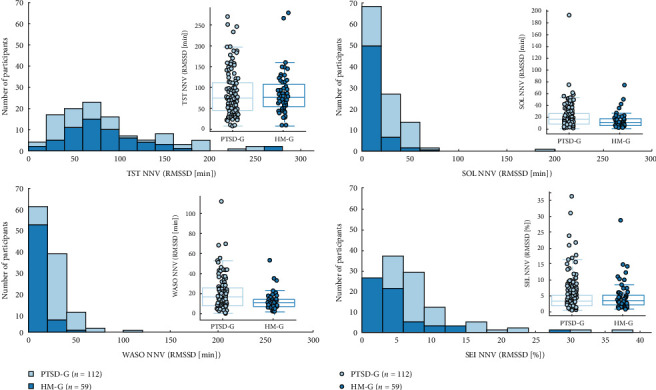
Night-to-night variability (NNV) in sleep parameters between PTSD-G (*n* = 112) and HM-G (*n* = 59). root mean squared successive differences (RMSSDs) is used as an index. The histograms and accompanying box plots highlight the differences in variability between both PTSD-G and HM-G for each sleep parameter. *⁣*^*∗∗*^*p*  < 0.01 and *⁣*^*∗∗∗*^*p*  < 0.001, statistical differences between the two groups.

These findings are illustrated in Figure 3.

### 4.4. Physiological and Clinical Correlates

**Figure 4:**
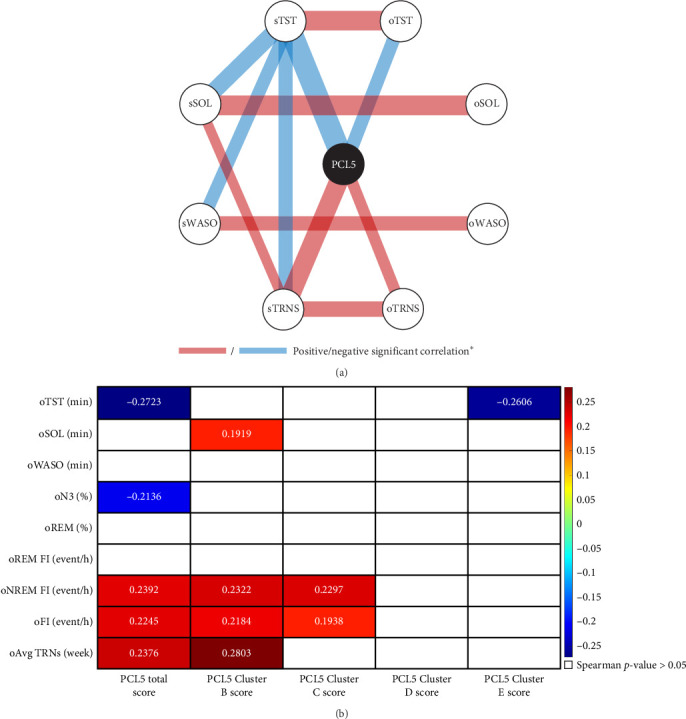
(A) Network analysis. *⁣*^*∗*^Line's width represents significant Spearman's rank correlation coefficient. *s*-parameters correspond to subjective parameters from the Pittsburgh sleep quality index (PSQI). The *o*-parameters correspond to objective parameters obtained with the DH. (B) Spearman correlation matrix between objective sleep parameters and PCL5 total score and symptom clusters (Cluster B reflects intrusive symptoms; Cluster C, avoidance symptoms; Cluster D, negative alterations in cognition and mood; and Cluster E, alterations in arousal and reactivity). Only statistically significant correlations are displayed. FI = fragmentation index (events per hour); %N3 = Stage 3 sleep, percentage of total sleep; PCL5 = post-traumatic stress disorder checklist for DSM-5, total score; %REM, percentage of total sleep time spent in rapid eye movement sleep; oTST, objective TST; SOL, sleep onset latency; TST, total sleep time; WASO, wake after sleep onset.

The analysis revealed significant correlations between objective and subjective sleep parameters, demonstrating the consistency of home-recorded sleep data. A positive correlation was found between objective and subjective TST (Spearman's *ρ* = 0.3860, *p*  < 0.001), SOL (Spearman's *ρ* = 0.4069, *p*  < 0.001), and WASO (Spearman's *ρ* = 0.2070, *p*=0.031), as shown in Figure 4A. Additionally, a significant positive association was identified between the objective frequency of TRNS reported via E4 push-button and the subjective frequency reported in the TRNS-FR questionnaire (Spearman's *ρ* = 0.3661, *p*  < 0.001).

Conversely, objective SOL (oSOL) was positively correlated with PCL-5 Cluster B (intrusion; *ρ* = 0.1919, *p*=0.0498), indicating that individuals with more frequent intrusion symptoms took longer to fall asleep. Similarly, the frequency of TRNs was positively correlated with PCL-5 total score (*ρ* = 0.2376, *p*=0.04764) and PCL-5 Cluster B (intrusion; *ρ* = 0.2803, *p*=0.01876), further reinforcing the link between intrusive symptoms and disrupted sleep continuity. The FI showed positive correlations with the PCL-5 Total Score (*ρ* = 0.2245, *p*=0.022), PCL-5 Cluster B (intrusion; *ρ* = 0.2184, *p*=0.0252), and PCL-5 Cluster C (avoidance; *ρ* = 0.1938, *p*=0.0476), suggesting that greater PTSD severity, particularly in the domains of intrusion and avoidance, was associated with increased sleep discontinuity. These results are illustrated in Figure 4B. Following statistical corrections, the only statistically significant finding was a negative correlation between oTST and the PCL-5 total score (*ρ* = −0.27227, *p*_adj = 0.041377).

## 5. Discussion

### 5.1. Clinical Characteristics and Sleep Macrostructure of PTSD-Related Sleep Disturbances

This study highlights sleep disturbances specifically associated with PTSD and examines their manifestations in a population of military service members, revealing both clinical presentations and measurable alterations in sleep macrostructure. Nearly all patients in the PTSD group reported significant sleep complaints, with 97.64% experiencing insomnia (mean ISI score of 18.47), 84.25% suffering from TRNs, and 70.08% with prolonged SOL. Additionally, 64.23% were classified as high risk for OSA, further exacerbating sleep fragmentation and its impact on daytime PTSD symptoms [40]. These disturbances are in line with findings linking PTSD-related sleep disruptions to symptoms of hyperarousal and intrusion, which interfere with both sleep initiation and continuity [4, 10]. Additionally, the presence of significant depressive symptoms in nearly all patients (98.37%), with 35.77% classified as severe, may further amplify sleep fragmentation and daytime symptoms of PTSD [2]. This is a worrying factor, as distressing dreams and sleep disturbances, and the replicative quality of trauma nightmares have been shown to be strongly associated with suicidal ideation, particularly in trauma-exposed individuals [41].

In accordance with our primary objective, we characterized sleep disturbances in PTSD patients and compared their home-recorded sleep patterns with those of healthy military controls. Surprisingly, despite patient complaints about reduced sleep duration, objective TST (oTST) did not significantly differ between groups. This finding might be surprising, as shorter TST is reported in combat-exposed PTSD veterans compared with non-PTSD veterans, in a laboratory PSG sleep assessment [17]. In comparison, our ecological recording methodology captured real-life sleep patterns, including individual adaptations such as extended time in bed due to sick leave (70.87%) and compensatory sleep habits, as well as the influence of psychotropic medications (e.g., neuroleptics reducing N3 sleep and antidepressants suppressing REM sleep), which may have masked differences in TST [8, 14].

Nevertheless, our results confirm the existence of significant sleep disturbances in PTSD patients, which is consistent with previous research [14]. Increased SOL, greater WASO, and reduced SEI are in line with previous meta-analyses [14]. Additionally, PTSD patients exhibited shorter N3 and REM sleep durations, further reflecting disruptions in sleep architecture [14]. While the exact mechanisms remain uncertain, some studies suggest that alterations in REM sleep may disrupt emotional memory processing, thereby, sustaining intrusive phenomena such as TRNs [18, 25]. Importantly, this relationship may be bidirectional, as TRNs themselves could further fragment sleep, reinforcing disrupted sleep continuity in PTSD [2, 25]. Similarly, reduced N3 sleep, which plays a role in homeostatic regulation and emotional resilience, may contribute to greater sleep instability.

After adjusting for age and psychotropic use, only SOL, wake duration, SEI, and N3 sleep latency remained significant, reinforcing their relevance as key markers of PTSD-related sleep alterations [14]. However, unadjusted results remain valuable as they reflect real-life sleep patterns, despite lower specificity. Over-adjustment is a concern [42], as the most severe cases of PTSD—often characterized by greater sleep fragmentation and deficits in fear extinction [2, 9]—are also more likely to receive psychotropic treatments. Adjustments may, therefore, remove variance intrinsically linked to PTSD severity, which could underestimate its true impact on sleep. By presenting unadjusted and adjusted analyses, we provide a comprehensive view that accounts for PTSD-specific sleep alterations while considering external influences on sleep architecture and symptom persistence [18, 25].

### 5.2. Within-Group and Night-to-Night Sleep Variability (WGV and NNV)

A secondary objective of this study was to assess WGV (over time within a group) and NNV (from night to night) in sleep parameters. Beyond the higher duration of sleep latency (SOL) and disrupted sleep continuity and efficiency (higher WASO and lower SEI) in PTSD-G compared to HM-G, all three parameters show greater dispersion and a higher frequency of outliers, reflecting significant instability of sleep patterns. This heterogeneity suggests that, even in individuals with comparable diagnoses of PTSD, sleep disturbances manifest differently, likely influenced by variations in trauma exposure, coping strategies, comorbid conditions, and symptom severity [12, 43, 44].

NNV was also more pronounced in the PTSD-G, with significant fluctuations observed for SOL, WASO, and SEI, aligning with research showing greater fluctuations in sleep among PTSD patients compared with patients with primary insomnia or healthy controls [23]. Notably, SOL was not only almost twice as long on average in the PTSD-G but also displayed substantial NNV, underscoring the need to assess fluctuations (through RMSSD analysis) rather than relying solely on mean values. In contrast, TST NNV did not differ significantly between groups, suggesting that fluctuations in sleep initiation and maintenance, rather than total sleep duration, are more relevant to PTSD-related sleep disruptions.

Beyond individual differences, these findings highlight the heterogeneity of PTSD-related sleep disturbances. Even within a homogeneous veteran sample, internal and external factors—such as symptom severity, TRNs occurrence, and coping strategies—are likely to contribute to variations in sleep patterns. This variability is expected to be even greater in studies including mixed-sex samples and diverse trauma types, complicating direct comparisons across populations [11, 17–19]. A key factor in this heterogeneity is the heightened environmental sensitivity of PTSD patients, which may partially explain the pronounced NNV observed in SOL, WASO, and SEI. These variations have been linked to daily fluctuations in stress exposure, trauma-related cues, and autonomic reactivity, with sleep instability closely associated with increased daytime symptom severity, reinforcing the bidirectional relationship between PTSD and sleep disturbances [2, 3, 14].

These findings highlight the limitations of single-night recordings and static averages in capturing the complexity of PTSD-related sleep disturbances. Although multinight assessments offer a broader perspective, averaging across nights may still mask key fluctuations due to symptom intensity, trauma-related cues, or environmental factors [3, 5, 23, 25]. Pronounced NNV suggests that relying solely on mean values could underestimate the extent of sleep fragmentation and instability. Future studies should incorporate dynamic assessment methods, such as ecological momentary assessment (EMA) or daily sleep monitoring, to better relate nocturnal variability to daily symptomatology and external triggers [9].

### 5.3. Relationship Between Sleep Disturbances and PTSD Symptoms (PCL-5 Clusters)

Finally, we hypothesized that sleep fragmentation, prolonged WASO and SOL, and reduced TST would be linked to intrusion (Cluster B) and hyperarousal (Cluster E) symptoms. However, our findings did not support this, as PTSD severity correlated primarily with TST, N3 percentage, and sleep fragmentation, particularly in NREM sleep. Moreover, fragmentation was more strongly associated with intrusion (B) and avoidance (C) than hyperarousal (E).

Although previous studies have emphasized REM sleep disturbances as a hallmark of PTSD [14, 15], the lack of significant correlation between REM fragmentation and PTSD severity in our study suggests that its role may be more prominent in the early stages of PTSD rather than in the persistence of symptoms. Instead, NREM fragmentation appears more directly related to symptom intensity, potentially playing a more important role in the ongoing modulation of symptoms. One possible explanation is that disruptions in deep sleep (N3), which plays an important role in emotional memory consolidation and fear extinction, may contribute to impaired downregulation of traumatic memories and increased autonomic instability [16]. Given that NREM accounts for around 75% of the sleep cycle, trauma-related memories are more frequently activated during this phase. Fragmented N3 may prevent the integration of trauma-related memories into a broader autobiographical context, thereby, reinforcing intrusive recollections and maladaptive avoidance behaviors. Moreover, the strong interaction between sleep continuity and the autonomic nervous system suggests that NREM instability may exacerbate nighttime hyperarousal, further disrupting sleep and perpetuating avoidance strategies [2]. In this context, the significant correlation between the number of TRNs per night (through the E4 Empatica wristband) and Cluster B symptoms of the PCL-5 aligns with previous findings showing that TRNs predominantly occur in NREM sleep, particularly early in the night [24, 25]. This supports the hypothesis that trauma re-experiencing in PTSD is closely linked to the reactivation of episodic memory related to NREM, further destabilizing sleep and reinforcing PTSD symptomatology [45].

Although the multiple correlations did not remain significant after correction for multiple comparisons, this does not diminish their clinical relevance. The complexity of PTSD-related sleep disturbances, shaped by many interacting factors, probably explains this attenuation. Notably, the strongest associations—such as the link between reduced oTST and PTSD severity—remained significant, reinforcing the central role of sleep disruptions in PTSD symptomatology.

### 5.4. Clinical and Research Implications: Personalized Approaches and Long-Term Recording

The high NNV in sleep patterns observed in PTSD patients highlights the importance of sleep stabilization as a therapeutic target. Poor sleep regularity has been associated with increased PTSD symptom burden, suggesting that interventions aimed at reducing sleep variability—rather than treating hyperarousal alone—may be particularly beneficial [23, 46]. Cognitive behavioral therapy for insomnia (CBT-I) has shown efficacy in populations with PTSD, improving objective and subjective sleep parameters [47]. However, given the pronounced inter-individual variability in sleep architecture, standardized approaches may not be optimal for all patients. This is particularly true in military populations, where disrupted sleep patterns are highly individual and often resistant to conventional interventions [46, 47].

To address this heterogeneity, a two-tiered approach may be warranted. First, interventions focused on sleep stabilization—such as strict sleep–wake schedules, sleep restriction therapy, and targeted psychoeducation—could mitigate night-to-night fluctuations in sleep continuity [16, 46]. Second, tailored strategies should be developed to target specific sleep phenotypes. For example, patients with persistent nightmares may benefit from interventions addressing both TRNs and sleep fragmentation. Our findings reinforce the strong link between NREM fragmentation and PTSD severity, consistent with evidence that TRNs predominantly occur during NREM sleep [24, 25]. This suggests that reducing nightmare frequency may contribute to stabilize sleep architecture and alleviate PTSD symptoms.

Various therapeutic strategies have been proposed to target nightmares, including imagery rehearsal therapy (IRT) and prazosin, both of which have been shown effective in reducing nightmare frequency and severity. Additionally, wearable devices such as NightWare leverage physiological markers to detect hyperarousal during TRNs and provide haptic feedback to interrupt the nightmare cycle without waking the sleeper [48].

Furthermore, the observed significant correlations between subjective and objective sleep parameters highlight the clinical relevance of self-reported sleep disturbances in PTSD patients. These correlations support the use of subjective assessments as valuable tools to track sleep disruptions and guide treatment, reinforcing their role in routine patient follow-up. From a research perspective, integrating EMA with wearable EEG monitoring and other modern sleep-tracking technologies could refine the characterization of sleep phenotypes in PTSD, helping to untangle the complex interactions between daily symptom fluctuations, autonomic reactivity, and sleep disturbances [12, 46]. Such an approach would enable real-time monitoring of symptom severity and sleep dynamics, facilitating a more individualized understanding of PTSD-related sleep pathology. This could in turn inform the development of personalized treatment pathways, ensuring that interventions are tailored to specific sleep phenotypes and patient needs. Future studies should determine whether timing therapeutic interventions to coincide with periods of improved sleep stability enhances treatment efficacy and long-term PTSD outcomes [49].

### 5.5. Limitations

Although home-based sleep recordings using wearable EEG devices provide ecologically valid assessments, several limitations must be acknowledged. First, although the DH has been validated in healthy populations, its accuracy in PTSD remains uncertain. PTSD-related sleep disturbances, such as frequent arousals, night sweats, and REM fragmentation, may introduce artifacts affecting sleep stage classification. While studies have demonstrated moderate-to-strong concordance between Dreem and PSG in clinical conditions such as mood disorders, epilepsy, and postconcussion syndrome [50], further validation in PTSD is needed, particularly to refine automated scoring algorithms.

Second, data loss due to technical issues and poor EEG signal quality remains a challenge in ambulatory sleep research. In the PTSD group, 111 nights (17.08% of the theoretical 650 nights) were lost due to technical issues and 84 nights (12.92%) were excluded due to a score index below 80%, leaving 455 nights available for analysis (70.00%). In the control group, 22 nights (6.77% of 325) were lost to technical issues and 28 nights (8.61%) were excluded due to poor signal quality, leaving 275 nights available (84.62%). Despite these exclusions, our dataset remains robust, covering 86.15% of PTSD patients and 90.77% of controls.

Another limitation is the lack of daily assessments of PTSD symptom. The PCL-5 was administered only at inclusion and at study completion, which precluded a fine-grained analysis of symptom fluctuations in relation to sleep variability. Future studies should incorporate EMA and daily symptom diaries to better capture dynamic interactions between sleep and PTSD symptoms.

Psychotropic medications also deserve consideration, as antidepressants, antipsychotics, and benzodiazepines influence sleep architecture, including suppression of REM sleep and alterations of deep sleep [51]. To address this issue, we controlled for psychotropic use, but avoided overadjustment by presenting both unadjusted and adjusted analyses to ensure transparent interpretation. Additionally, age differences between the PTSD and control groups reflect the demographic composition of active-duty military personnel. Although age-adjusted analyses did not change the main findings, this remains a methodological consideration for generalizability.

Finally, although our five-night recording period exceeds standard PSG protocols, it may not fully reflect long-term variability of sleep disturbances. Given the influence of external stressors and daytime symptom burden on PTSD-related sleep disruptions, future research should extend monitoring periods and incorporate longitudinal recordings for a more comprehensive assessment.

### 5.6. Conclusion

This study demonstrates the utility of ecologically valid, multinight sleep recording in military personnel with PTSD, revealing significant NNV in sleep disturbances that is closely related to PTSD symptom severity. These findings highlight the importance of personalized longitudinal assessments to inform targeted interventions that address fluctuations in sleep patterns and associated symptoms. This approach paves the way for advanced research on sleep as a modifiable factor in PTSD prognosis, with direct implications for improving clinical outcomes through personalized therapeutic strategies.

## Figures and Tables

**Table 1:** Sociodemographic and clinical characteristics of PTSD patients and healthy military group.

Characteristics	PTSD-G (*N* = 130)	HM-G (*N* = 65)	*p*-value
Sociodemographic			
Age (years)	39.95 (9.30)	35.20 (8.93)	**0.002**
Number of deployments	5.15 (4.45)	4.63 (3.71)	0.651
Number of traumas	7.07 (11.71)	2.61 (4.65)	**<0.001**
Clinical			
PTSD	—	—	—
PCL-5	46.72 (12.78)	3.37 (8.19)	**<0.001**
IES-R	55.74 (14.89)	4.14 (6.64)	**<0.001**
Depression			
BDI total score	13.79 (5.53)	1.51 (2.24)	**<0.001**
Sleep complaints at the time of inclusion			
Yes	97.64%	30.77%	**<0.001**
No	2.36%	69.23%	**<0.001**
Sleep complaints			
Nightmares	84.25%	9.23%	**<0.001**
Sleep onset latency	70.08%	13.85%	**0.018**
Early awakening	40.16%	4.62%	**0.026**
Nocturnal awakenings	84.25%	12.31%	**<0.001**
Ineffective sleep	66.14%	9.23%	**0.001**
Insomnia			
ISI total score	18.47 (3.68)	3.31 (2.84)	**<0.001**
Sleep Quality			
PSQI total score	12.43 (3.68)	4.24 (1.82)	**<0.001**
Subjective TST (min)	331.07 (100.80)	426.46 (56.56)	**<0.001**
Subjective SOL (min)	57.02 (43.52)	18.23 (12.58)	**<0.001**
PSQI-A	14.70 (5.01)	0.70 (1.80)	**<0.001**
OSA			
Berlin questionnaire total score	1.71 (0.80)	0.57 (0.71)	**<0.001**
BMI	27.94 (4.46)	25.60 (4.49)	**<0.001**
High risk of OSA	64.23%	12.31%	**<0.001**
Sleepiness			
ESS total score	10.35 (5.63)	5.94 (4.31)	**<0.001**

*Note*: Values are presented as mean (SD) or percentage (%). Results that remain significant after adjustment for multiple comparisons using the Benjamini–Hochberg procedure are highlighted in bold.

Abbreviations: BDI, Beck depression inventory; ESS, Epworth sleepiness scale; IES-R, impact of event scale; ISI, insomnia sleep index; PCL-5, PTSD checklist for DSM-5; PCL-S, post-traumatic stress checklist scale; PSQI, Pittsburgh sleep quality index; PSQI-A, PSQI addendum for PTSD.

**Table 2:** Comparison of sleep parameters between the PTSD group (PTSD-G, *N* = 112) and the healthy military group (HM-G, *N* = 59).

Sleep parameters	PTSD-G (*N* = 112)	HM-G (*N* = 59)
	Mean (SD)	

Sleep parameters		
TST (min)	396.94 (71.74)	399.79 (53.31)
SOL (min)	**30.71** (21.78)*⁣*^*∗∗∗*^	16.60 (8.82)
WASO (min)	**31.95** (20.87)*⁣*^*∗∗∗*^	22.79 (19.70)
SEI (%)	**85.73** (7.06)*⁣*^*∗∗∗*^	92.34 (4.05)
Wake (min)	**65.88** (32.83)*⁣*^*∗∗∗*^	41.21 (19.50)
Sleep stages		
N1—duration (min)	29.45 (11.98)	27.03 (8.95)
N1 (%)	7.52 (3.31)	6.82 (2.06)
N2—duration (min)	202.01 (50.94)	186.94 (41.69)
N2 (%)	**50.68** (9.10)*⁣*^*∗∗*^	46.29 (6.69)
N3—duration (min)	**68.12** (32.56)*⁣*^*∗*^	76.69 (24.36)
N3 (%)	17.55 (7.88)	19.78 (7.11)
REM—duration (min)	**97.36** (34.40)*⁣*^*∗*^	109.12 (24.20)
REM (%)	**24.25** (6.95)*⁣*^*∗∗*^	27.11 (5.03)
NREM—duration (min)	299.58 (55.90)	290.57 (43.42)
NREM (%)	**75.75** (6.95)*⁣*^*∗∗*^	72.89 (5.03)
Sleep latencies		
N2 (min)	5.12 (5.20)	3.78 (2.20)
N3 (min)	**24.31** (16.66)*⁣*^*∗∗*^	19.17 (11.74)
REM (min)	**100.49** (44.46)*⁣*^*∗∗∗*^	74.18 (14.47)
Sleep fragmentation		
FI (events/h)	6.30 (2.63)	5.94 (1.59)
REM FI (events/h)	9.66 (3.80)	9.42 (2.74)
NREM FI (events/h)	6.34 (2.72)	5.78 (1.51)
Microarousal index	9.39 (4.35)	9.22 (3.61)

*Note:* Significant results are highlighted in bold.

*⁣* ^*∗*^ *p*  < 0.05, *⁣*^*∗∗*^*p*  < 0.01, and *⁣*^*∗∗∗*^*p*  < 0.001.

## Data Availability

The data that support the findings of this study are available from the corresponding author upon reasonable request.

## References

[1] Mysliwiec V., Gill J., Lee H. (2013). Sleep Disorders in US Military Personnel: A High Rate of Comorbid Insomnia and Obstructive Sleep Apnea. *Chest*.

[2] Saguin E., Gomez-Merino D., Sauvet F., Leger D., Chennaoui M. (2021). Sleep and PTSD in the Military Forces: A Reciprocal Relationship and a Psychiatric Approach. *Brain Sciences*.

[3] van Liempt S., van Zuiden M., Westenberg H., Super A., Vermetten E. (2013). Impact of Impaired Sleep on the Development of PTSD Symptoms in Combat Veterans: A Prospective Longitudinal Cohort Study. *Depression and Anxiety*.

[4] Rosen R. C., Cikesh B., Fang S. (2019). Posttraumatic Stress Disorder Severity and Insomnia-Related Sleep Disturbances: Longitudinal Associations in a Large, Gender-Balanced Cohort of Combat-Exposed Veterans. *Journal of Traumatic Stress*.

[5] Pigeon W. R., Campbell C. E., Possemato K., Ouimette P. (2013). Longitudinal Relationships of Insomnia, Nightmares, and PTSD Severity in Recent Combat Veterans. *Journal of Psychosomatic Research*.

[6] Neylan T. C., Marmar C. R., Metzler T. J. (1998). Sleep Disturbances in the Vietnam Generation: Findings From a Nationally Representative Sample of Male Vietnam Veterans. *Am J Psychiatry*.

[7] Taylor D. J., Pruiksma K. E., Hale W. (2020). Sleep Problems in Active Duty Military Personnel Seeking Treatment for Posttraumatic Stress Disorder: Presence, Change, and Impact on Outcomes. *Sleep*.

[8] Pruiksma K. E., Taylor D. J., Wachen J. S. (2016). Residual Sleep Disturbances Following PTSD Treatment in Active Duty Military Personnel. *Psychological Trauma: Theory, Research, Practice, and Policy*.

[9] Zayfert C., DeViva J. C. (2004). Residual Insomnia Following Cognitive Behavioral Therapy for PTSD. *Journal of Traumatic Stress*.

[10] Krakow B., Hollifield M., Johnston L. (2001). Imagery Rehearsal Therapy for Chronic Nightmares in Sexual Assault Survivors With Posttraumatic Stress Disorder: A Randomized Controlled Trial. *JAMA*.

[11] Mellman T. A., Kobayashi I., Lavela J., Wilson B., Hall Brown T. S. (2014). A Relationship Between REM Sleep Measures and the Duration of Posttraumatic Stress Disorder in a Young Adult Urban Minority Population. *Sleep*.

[12] Van Liempt S., Vermetten E., Lentjes E., Arends J., Westenberg H. (2011). Decreased Nocturnal Growth Hormone Secretion and Sleep Fragmentation in Combat-Related Posttraumatic Stress Disorder: Potential Predictors of Impaired Memory Consolidation. *Psychoneuroendocrinology*.

[13] Brownlow J. A., Miller K. E., Ross R. J. (2022). The Association of Polysomnographic Sleep on Posttraumatic Stress Disorder Symptom Clusters in Trauma-Exposed Civilians and Veterans. *SLEEP Advances*.

[14] Zhang Y., Ren R., Sanford L. D. (2019). Sleep in Posttraumatic Stress Disorder: A Systematic Review and Meta-Analysis of Polysomnographic Findings. *Sleep Medicine Reviews*.

[15] Mellman T. A., Bustamante V., Fins A. I., Pigeon W. R., Nolan B. (2002). REM Sleep and the Early Development of Posttraumatic Stress Disorder. *American Journal of Psychiatry*.

[16] Pace-Schott E. F., Germain A., Milad M. R. (2015). Sleep and REM Sleep Disturbance in the Pathophysiology of PTSD: The Role of Extinction Memory. *Biology of Mood & Anxiety Disorders*.

[17] Wang C., Laxminarayan S., Ramakrishnan S. (2020). Increased Oscillatory Frequency of Sleep Spindles in Combat-Exposed Veteran Men With Post-Traumatic Stress Disorder. *Sleep*.

[18] Germain A. (2013). Sleep Disturbances as the Hallmark of PTSD: Where Are We Now?. *American Journal of Psychiatry*.

[19] Pillar G., Malhotra A., Lavie P. (2000). Post-Traumatic Stress Disorder and Sleep-What a Nightmare!. *Sleep Medicine Reviews*.

[20] Woodward S. H., Arsenault N. J., Murray C., Bliwise D. L. (2000). Laboratory Sleep Correlates of Nightmare Complaint in PTSD Inpatients. *Biological Psychiatry*.

[21] Hurwitz T. D., Mahowald M. W., Kuskowski M., Engdahl B. E. (1998). Polysomnographic Sleep Is Not Clinically Impaired in Vietnam Combat Veterans With Chronic Posttraumatic Stress Disorder. *Biological Psychiatry*.

[22] Biggs Q. M., Ursano R. J., Wang J. (2019). Daily Variation in Post Traumatic Stress Symptoms in Individuals With and Without Probable Post Traumatic Stress Disorder. *BMC Psychiatry*.

[23] Straus L. D., Drummond S. P., Nappi C. M., Jenkins M. M., Norman S. B. (2015). Sleep Variability in Military-Related PTSD: A Comparison to Primary Insomnia and Healthy Controls. *Journal of Traumatic Stress*.

[24] Richards A., Woodward S. H., Baquirin D. P. G. (2023). The Sleep Physiology of Nightmares in Veterans With Psychological Trauma: Evaluation of a Dominant Model Using Participant-Applied Electroencephalography in the Home Environment. *Journal of Sleep Research*.

[25] Saguin E., Feingold D., Roseau J. B. (2023). An Ecological Approach to Clinically Assess Nightmares in Military Service Members With Severe PTSD. *Sleep Medicine*.

[26] Arnal P. J., Thorey V., Debellemaniere E. (2020). The Dreem Headband Compared to Polysomnography for Electroencephalographic Signal Acquisition and Sleep Staging. *Sleep*.

[27] Paul F., Pommier de Santi V., Marimoutou C., Deparis X. (2013). Validation de l’échelle PCLS et d’un auto-questionnaire court dans le cadre du dépistage des états de stress post-traumatiques chez les militaires de retour de mission. *Dans La psychiatrie en milieu militaire*.

[28] Ashbaugh A. R., Houle-Johnson S., Herbert C., El-Hage W., Brunet A. (2016). Psychometric Validation of the English and French Versions of the Posttraumatic Stress Disorder Checklist for DSM-5 (PCL-5). *PLOS ONE*.

[29] Hansenne M., Charles G., Pholien P., Panzer M., Pitchot W., Ansseau M. (1993). Mesure subjective de l’impact d’un événement: traduction française et adaptation de l’échelle d’Horowitz. *Psychol Méd*.

[30] Collet L., Cottraux J. (1986). Inventaire abrégé de la dépression de Beck (13 items). Étude de la validité concurrente avec les échelles de Hamilton et de ralentissement de Widlöcher. *L’Encephale*.

[31] Buysse D. J., Reynolds CF 3rd, Monk T. H., Berman S. R., Kupfer D. J. (1989). The Pittsburgh Sleep Quality Index: A New Instrument for Psychiatric Practice and Research. *Psychiatry Research*.

[32] Ait-Aoudia M., Levy P. P., Bui E. (2013). Validation of the French Version of the Pittsburgh Sleep Quality Index Addendum for Posttraumatic Stress Disorder. *European Journal of Psychotraumatology*.

[33] Saguin E., Hulot L. J., Roseau J. B. (2023). Cross-Cultural Adaptation and Preliminary Validation of a French Version of the Trauma-Related Nightmare Survey (TRNS-FR) in a PTSD Veteran Population. *Military Medicine*.

[34] Leger D., Guilleminault C., Dreyfus J. P., Delahaye C., Paillard M. (2000). Prevalence of Insomnia in a Survey of 12,778 Adults in France. *Journal of Sleep Research*.

[35] Khaledi-Paveh B., Khazaie H., Nasouri M., Ghadami M. R., Tahmasian M. (2016). Evaluation of Berlin Questionnaire Validity for Sleep Apnea Risk in Sleep Clinic Populations. *Basic and clinical neuroscience*.

[36] Johns M. W. (1991). A New Method for Measuring Daytime Sleepiness: The Epworth Sleepiness Scale. *Sleep*.

[37] van Lier H. G., Pieterse M. E., Garde A. (2020). A Standardized Validity Assessment Protocol for Physiological Signals From Wearable Technology: Methodological Underpinnings and an Application to the E4 Biosensor. *Behavior Research Methods*.

[38] Haba-Rubio J., Ibanez V., Sforza E. (2004). An Alternative Measure of Sleep Fragmentation in Clinical Practice: The Sleep Fragmentation Index. *Sleep Medicine*.

[39] Dancey C., Reidy J. (2004). *Statistics Without Maths for Psychology: Using SPSS for Windows*.

[40] McCall C. A., Watson N. F. (2022). A Narrative Review of the Association Between Post-Traumatic Stress Disorder and Obstructive Sleep Apnea. *Journal of Clinical Medicine*.

[41] Richards A., Santistevan A., Kovnick M. (2025). Distressing Dreams in Trauma Survivors: Using a Sleep Diary Mobile App to Reveal Distressing Dream Characteristics and Their Relationship to Symptoms and Suicidal Ideation in Trauma-Exposed Adults. *Sleep Advances*.

[42] Schisterman E. F., Cole S. R., Platt R. W. (2009). Overadjustment Bias and Unnecessary Adjustment in Epidemiologic Studies. *Epidemiology*.

[43] Levin R., Nielsen T. A. (2007). Disturbed Dreaming, Posttraumatic Stress Disorder, and Affect Distress: A Review and Neurocognitive Model. *Psychological Bulletin*.

[44] Mohsenin S., Mohsenin V. (2014). Diagnosis and Management of Sleep Disorders in Posttraumatic Stress Disorder: A Review of the Literature. *The Primary Care Companion For CNS Disorders*.

[45] Baylor G. W., Cavallero C. (2001). Memory Sources Associated With REM and NREM Dream Reports Throughout the Night: A New Look at the Data. *Sleep*.

[46] Remadi M., Dinis S., Bernard L. (2024). Evaluation of Sleep and Therapeutic Education Needs of Military With PTSD. *L’Encéphale*.

[47] Colvonen P. J., Straus L. D., Stepnowsky C., McCarthy M. J., Goldstein L. A., Norman S. B. (2018). Recent Advancements in Treating Sleep Disorders in Co-Occurring PTSD. *Current Psychiatry Reports*.

[48] Davenport N. D., Werner J. K. (2023). A Randomized Sham-Controlled Clinical Trial of a Novel Wearable Intervention for Trauma-Related Nightmares in Military Veterans. *Journal of Clinical Sleep Medicine*.

[49] Baddeley J. L., Gros D. F. (2013). Cognitive Behavioral Therapy for Insomnia as a Preparatory Treatment for Exposure Therapy for Posttraumatic Stress Disorder. *American Journal of Psychotherapy*.

[50] Markov K., Elgendi M., Menon C. (2024). EEG-Based Headset Sleep Wearable Devices. *NPJ Biosensing*.

[51] Ghossoub E., Geagea L., Kobeissy F., Talih F. (2021). Comparative Effects of Psychotropic Medications on Sleep Architecture: A Retrospective Review of Diagnostic Polysomnography Sleep Parameters. *Sleep Science (Sao Paulo, Brazil)*.

